# The air we breathe: Numerical investigation of ventilation strategies to mitigate airborne dispersion of MERS-CoV in inpatient wards

**DOI:** 10.1016/j.heliyon.2024.e26159

**Published:** 2024-02-14

**Authors:** Manoj Kumar Satheesan, Tsz Wun Tsang, Ling Tim Wong, Kwok Wai Mui

**Affiliations:** Department of Building Environment and Energy Engineering, The Hong Kong Polytechnic University, Hung Hom, Hong Kong SAR, China

**Keywords:** Airborne dispersion, Open ward cubicle, Ventilation, Infection control, Computational fluid dynamics, Nosocomial infection

## Abstract

Ventilation strategies for infection control in hospitals has been predominantly directed towards isolation rooms and operating theatres, with relatively less emphasis on perceived low risk spaces, such as general wards. Typically, the ventilation systems in general wards are intended to optimize patient thermal comfort and energy conservation. The emission of pathogens from exhalation activity, such as sneezing, by an undiagnosed infectious patient admitted to general wards, is a significant concern for infection outbreaks. However, the ventilation guidelines for general wards with respect to infection control are vague. This research article presents a numerical study on the effect of varying air change rates (3 h^−1^, 6 h^−1^, 9 h^−1^, 13 h^−1^) and exhaust flow rates (10%, 50% of supply air quantity) on the concentration of airborne pathogens in a mechanically ventilated general inpatient ward. The findings imply that the breathing zone directly above the source patient has the highest level of pathogen exposure, followed by the breathing zones at the bedside and adjacent patients close to the source patient. The dispersion of pathogens throughout the ward over time is also apparent. However, a key difference while adopting a lower ACH (3 h^−1^) and a higher ACH (13 h^−1^) in this study was that the latter had a significantly lower number of suspended pathogens in the breathing zone than the former. Thus, this research suggests high ventilation rates for general wards, contrary to current ventilation standards. In addition, combining a higher air change rate (13 h^−1^) with a high exhaust flow rate (50% of supply air) through a local exhaust grille dramatically reduced suspended pathogens within the breathing zone, further mitigating the risk of pathogen exposure for ward users. Therefore, this study presents an effective ventilation technique to dilute and eliminate airborne infectious pathogens, minimizing their concentration and the risk of infection.

## Highlights

• Investigated the airborne dispersion of MERS-CoV in an inpatient ward.
• Evaluated spatial and temporal bioaerosol distribution at users breathing height.
• Local exhaust grille near patients reduces airborne pathogen exposure.
• High ACH and high exhaust flow rate through local exhaust grilles are recommended.
• Role of ventilation strategies in infection control is indisputable.

## 1 Introduction

Nosocomial infections, often called healthcare-associated infections (HAIs), are illnesses caused by contact with a healthcare environment and associated procedures [1]. These infections, which can affect patients, healthcare professionals, and visitors, can be caused by transmitting bacteria, viruses, fungi, or other pathogens. It can lengthen hospital stays, increase hospital expenditures, and increase patient morbidity and mortality [2]. At times, nosocomial infections may also cause massive outbreaks involving numerous patients and staff members, raising the risk of transmission and aggravating the infection's effects on the healthcare system [3]. Thus, preventing and managing nosocomial infections should be a critical priority in healthcare facilities.

The three widely recognised infection transmission routes are airborne, droplet and contact [4]. Airborne transmission is one of the principal mechanisms for Coronavirus diseases 2019 (COVID-19) [5,6], Middle-East Respiratory Syndrome-associated coronavirus (MERS-CoV) [7], and the SARS virus (2003) [8], all of which have caused catastrophic harm to human health and life on a global scale. Due to the potential for infectious pathogens to spread through the air, preventing airborne transmission in general wards is a significant problem [9], especially in settings where numerous patients are housed in proximity. General wards with a high patient population and frequent interactions between patients, healthcare personnel, and visitors have a higher chance of transmission [10,11]. Moreover, insufficient ventilation and poor air filtering would elevate infection transmission risk [12,13]. In these circumstances, ventilation's role as a passive approach to preventing infection transmission within healthcare facilities becomes increasingly significant.

Increased air change rates can expeditiously eliminate infectious airborne particles, minimizing the likelihood of exposure and transmission. It has been found to lessen the possibility of airborne viral transmission, particularly those responsible for respiratory diseases. This view, however, was called into doubt by a few studies that demonstrated that, under certain conditions, boosting the air change rate increased the probability of pathogen exposure risk [14,15]. Increasing air supply rates can help dilute indoor air contaminants, but it is not always sufficient for preventing the spread of infectious diseases through the air. Additional aspects to consider are air circulation, room arrangement, and filtration [[16], [17], [18]]. In addition to ACH, the design of a ventilation system is a regulating factor for pollutant flow routes [16,19,20].

Ren et al. [21] indicated that exhaust location could be crucial in removing contaminants in a mechanically ventilated COVID-19 inpatient ward. COVID-19 inpatient wards implement more rigorous and stringent ventilation protocols in comparison to general hospital wards. Nielsen et al.'s [22] study in a two-bed hospital ward with downward ventilation also indicated the importance of return openings on the contaminant distribution. This study utilized a tracer gas to simulate the gaseous contaminants under different air change rates and different postures of the patients. Tracer gas is frequently utilized to mimic the dispersion of droplet nuclei and assess the degree of airborne transmission. Nevertheless, its application is subject to certain constraints when compared to the use of particles [23,24]. The impact of exhaust location on the dispersion of contaminants in an operation theatre with an air exchange rate of 30 h^−1^ was investigated by Agriman et al. [25]. The study demonstrated that the implementation of both ceiling-level and floor-level exhaust systems can effectively decrease particle deposition onto the surgical table. Thatiparti et al. [26] underscored the importance of the pathway of contaminant flow from the source to the exhaust in a simulated airborne infection isolation room (AIIR). The significance of the exhaust's placement is deemed critical. Nevertheless, the influence of exhaust flow rates on the dispersion of contaminants remains inadequately examined.

Most of the research pertaining to the improvement of infection control in a clinical environment is focused on specialized facilities, such as isolation rooms and operating theatres. The ventilation strategies for these facilities are well characterised to mitigate airborne transmission of infection compared to general wards [27]. The specification of ward ventilation systems primarily focuses on ensuring patient comfort and reducing energy expenses, rather than being driven by clinical justifications. Therefore, the guidelines pertaining to the ventilation of general ward are relatively limited and frequently ambiguous in character [27]. Specialized facilities are subject to distinct ventilation design specifications, which differ from those of general wards [21]. For instance, the isolation room must be kept under negative pressure, whereas general wards are not subject to such stringent regulations. In addition, these specialist facilities adopt a higher ventilation rate in comparison to standard hospital wards [28,29]. Despite the evidence indicating the potential benefits of high ventilation rates in terms of pathogen dilution, the air change rate observed in general wards remains relatively low [27,30]. Thus, there is a need to reconsider the ventilation rates for presumed low-risk zones, such as a general inpatient ward based on evidence. However, it is imperative to exercise caution when formulating ventilation strategies for general wards based on insights gleaned from the study of specialized facilities. The aim of this investigation is to examine the interplay between the air change rate, exhaust location, and exhaust flow rate on the dispersion of infectious pathogens through the air in a typical inpatient ward environment.

Developing an effective ventilation strategy to reduce the risk of infection transmission would substantially benefit from exact and reliable numerical simulation models. The three methods used to predict the air distribution within a building are multi-zonal models [31], zonal models [32], and Computational fluid dynamics (CFD) [33]. Multi-zone models are a low-cost computational method; however, they consider each room a single node, limiting their accuracy and usefulness. Zonal approaches are viewed as a bridge between CFD and multi-zone models. CFD divides the computational area into several control volumes to comprehensively describe the fluid properties and solves the Navier-Stokes equations numerically. The accuracy is also good when comparing the results to multi-zone or zonal approaches [34]. Nonetheless, knowledgeable users who adhere to best practices recommendations are partially responsible for the results' reliability and correctness. As one of the most popular numerical simulation techniques, CFD has been used in various industries, including aircraft, manufacturing, buildings, and more [34]. CFD has emerged as a crucial technique for creating, analyzing, and evaluating the physical and functional configurations related to an indoor environment because of the rapid improvements in computing [35,36]. CFD simulations can offer a high spatial and temporal resolution of flow patterns, temperature, and contaminant dispersion within the computational domain of interest.

This study investigates the airborne dispersion of MERS-CoV in a general inpatient ward cubicle to propose an effective ventilation strategy to reduce the airborne spread of the viruses. By numerical simulation conducted with the CFD technique, the spatial and temporal dispersion of the virus under varying air change and exhaust flow rates is explored.

## 2 General inpatient ward

The inpatient facilities would vary by hospital type and patient need. Nonetheless, the most popular variants include the open ward, semi-private room, private room, isolation rooms, and intensive care unit. In hospitals, inpatient wards often use a considerable amount of floor area [37]. This study will focus on developing an effective ventilation strategy for the open ward design. In an open ward, many beds are arranged in a large space without partitions. This design is utilized frequently at Hong Kong's public hospitals and is intended to accommodate many patients cost-effectively [38]. Patients have access to shared restrooms and showers, and nurses are typically stationed nearby to administer treatment and monitor patients' status. Although open wards are less private than other inpatient facilities, they offer some benefits. They can provide patients with a sense of camaraderie and support by allowing them to interact with others in similar circumstances. Open wards also make it easier for nursing personnel to observe patients and address any difficulties that develop. There are, however, significant disadvantages to the open ward concept. Patients may be exposed to increased noise and disturbances from other patients and may have less privacy. In addition, there may be a more considerable risk of infection transmission in an open ward because patients are closer [39].

**Fig. 1:**
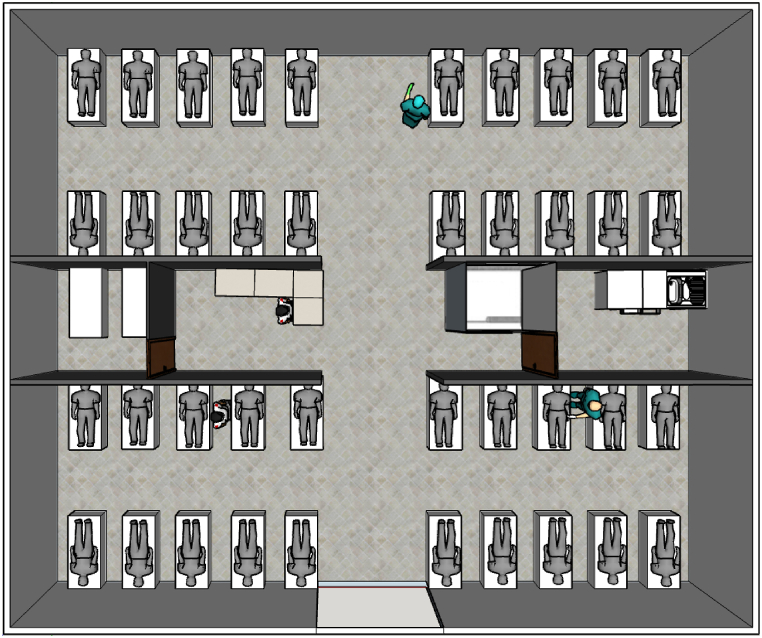
Representative image of general inpatient ward with four cubicles.

Fig. 1 is a representative layout of the general ward in Hong Kong during the outbreak of severe acute respiratory syndrome (SARS) [39]. The ward had four patient cubicles, a storage area, and a nurse station. It had central air conditioning, and a corridor separated the four semi-enclosed cubicles. The fan coil unit brought in fresh air from the outside and combined it with the air that had already been circulated inside the facility. The combination of fresh air with recirculated air that forms the supply air is then provided to the facility through a four-way supply air diffuser affixed on a false ceiling. In addition, the fan coil unit received recycled air from the corridor's exhaust grille.

**Fig. 2:**
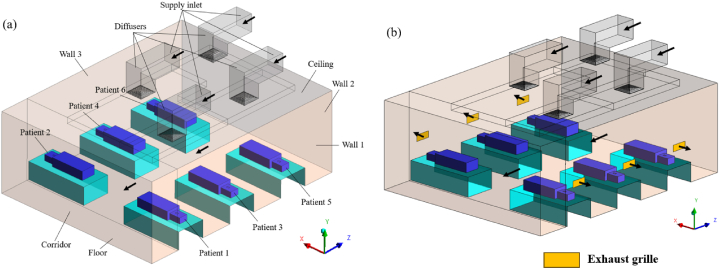
Inpatient ward with patients (a) No exhaust grille (b) Local exhaust grille.

A standard inpatient ward cubicle with dimensions of 7.5 m (L) x 6 m (W) x 2.7 m (H) was used in this study. Four supply diffusers measuring 0.6 m × 0.6 m were installed on the ceiling as part of the current ward design (base scenario), as illustrated in Fig. 2 (a). Mechanical ventilation was used that maintains a positive pressure toward the corridor in the six-bedded ward cubicle. In the same ward layout, our earlier research assessed the transport, dispersion, and deposition of MERS-CoV droplet nuclei [33]. Unfortunately, situations about exposure to infection through the deposition of droplet nuclei were only considered in that study. While contact transmission remains an important pathway for the spread of many infectious diseases, the airborne route must be prioritized while developing ventilation strategies. Hence, this study investigates the effectiveness of the ventilation strategy in mitigating exposure to infection through inhaling the particles suspended in the breathing zone air of the facility.

To overcome the constraints of our earlier work [33], the present study aims to investigate a more inclusive ventilation design that would mitigate the spread of infection through the airborne route in an inpatient hospital cubicle. Four air change rates (3 h^−1^, 6 h^−1^, 9 h^−1^, and 13 h^−1^) were adopted to evaluate the influence of ventilation rate on infection spread. Based on these air change rates, the air was supplied to the cubicle using ceiling-mounted diffusers. Local exhaust grilles were installed near the patient bed at each air change rate condition, as shown in Fig. 2 (b), regulated at 10% and 50% of the supply air. In the existing ward design, the ratio of supply air to ward air expelled to the corridor was maintained equally. In situations with exhaust grilles, the residual indoor air in the ward was vented to the corridor. Exhaust grilles were installed to investigate the effect of design changes on airflow and pollutant distribution in ward cubicles.

## 3 Methodology

### 3.1 Numerical simulation

Computational fluid dynamics is a powerful numerical simulation tool that can be used to model airflow distribution and particle transport within an inpatient ward environment. It utilizes several numerical schemes and algorithms to solve the governing equation of fluid flow and particle transport. It is primarily based on the finite volume method, a numerical scheme that divides the computational domain into numerous grids of cells and discretizes the governing equation over these cells. A multiphase numerical modelling technique similar to our earlier work was adopted in this study to evaluate the airflow distribution and particle transport within an inpatient ward cubicle [33]. The continuum phase constituted the air in the ward, and infectious pathogens expelled through sneezing were treated as a discrete phase. The Eulerian framework was utilized to solve the governing equations of continuity, momentum, and energy for the continuum phase, whereas the discrete phase was modelled in a Lagrangian framework. The three-dimensional incompressible turbulent airflow was modelled in a steady state and turbulence associated with indoor airflow was modelled using the Reynolds-averaged Navier Stokes (RANS) equation. In the RANS approach, the time-averaged Navier Stokes equation is solved. While doing the time-averaging procedure, unknown Reynolds stress tensors are created in equations, resulting in closure problems. The closure issue is addressed by utilizing the Eddy viscosity turbulence modelling. The renormalization group (RNG) k-ε is the most preferred eddy viscosity turbulence model to simulate the indoor airflow distribution [40,41]. The discretization of the governing equations was carried out using a second-order upwind scheme, and the pressure-velocity coupling in the continuum phase was accomplished through the utilization of the SIMPLE algorithm. A thermal boundary condition of constant heat flux was applied uniformly to all surfaces of the supine patients. With the exception of the walls consisting of heat sources in the form of patients, all remaining walls were considered adiabatic. The smooth no-slip condition has been implemented at all wall boundaries. The utilization of the Boussinesq approximation was implemented to mitigate the intricacy of modeling that arises from variations in density caused by temperature gradients.

**Fig. 3:**
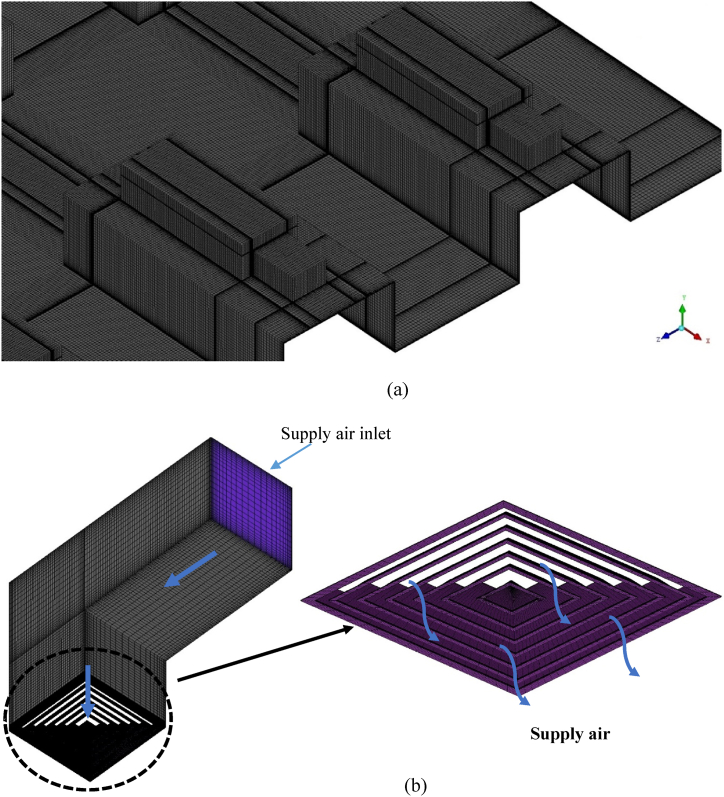
Grid generated within the computational domain (a) Thermal manikin on the bed (b) Supply air diffuser and flow deflector vanes (inset).

A grid generation tool, ICEM CFD, generated a hexahedral mesh with a grid spacing of 1.2 and a first cell height of 0.001 m from the computational wall. Fig. 3 (a, b) depicts two examples of the grid generated within the computational domain. The mesh in the proximity of the wall was refined to a degree that allowed for the resolution of the viscous sublayer (y+ < 5). The enhanced wall treatment strategy was used for near-wall modelling. Three grid systems namely, 1002k (System 1), 3202k (System 2), and 5110k (System 3) were generated using the grid generation tool. The results from airflow simulations done on each grid system were used to analyse the grid convergence using the grid convergence index (GCI) using Equation (1) [42,43]. It is computed by estimating the root-mean-square of the relative error (*e*_*rms*_) for mean fluid flow velocities (*u*) at 100 points along a vertical line at the centre of the cubicle as shown in Equation (2).

(1) $$G\;C\;I\left(u\right)=F_{s}\frac{e_{rms}}{r^{p}-1}$$

(2) $$e_{rms}=\sqrt{\frac{\Sigma_{m=1}^{100}{\left|\left(u_{m,coarse}-u_{m,fine}\right)/u_{m,fine}\right|}^{2}}{100}}$$

(3) $$r={\left(\frac{N_{fine}}{N_{coarse}}\right)}^{1/3}$$

In Equation (3), *r* is the refinement factor, which is determined by dividing the control volumes of the fine and coarse grid systems, *p* is the order of the discretization method and *F*_*s*_ is the safety factor. The GCIs of systems 2 and 3 were 3.11% and 3.40% using system 1 as reference [33]. Hence, System 2 was selected to investigate further fluid flow characteristics based on its computational efficiency and solution precision.

Violent expiratory episodes such as sneezing produce droplets that would evaporate rapidly. Considering this fact, the evaporation phenomenon was not modelled in this study. And in turn, their dried-out residual, also known as droplet nuclei, was modelled as a spherical particle with a size of 0.167 μm [33,44]. According to the conventional infection theory, bioaerosol particles that have a diameter less than 5 μm tend to remain suspended in the air and can be controlled through ventilation. Conversely, particles with larger diameters are believed to settle out of the air close to the source [45]. Furthermore, specific forces such as pressure gradient, virtual mass, and basset force are negligible in the indoor environment due to the size and density of droplet nuclei [46]. Although, drag, Brownian, thermophoretic, and Saffman lift forces were modelled to account for the size of the droplet nuclei and non-isothermal environment [33]. Table 1 provides further information on the CFD simulation settings used in this research. The authors of this study have maintained residual value 10^−6^ for energy and 10^−4^ for all other variables. Once the residuals fall below this tolerance, the solution is considered to have converged.

**Table 1:** CFD simulation parameters.

Computational domain	7.5 m(*L*) × 6 m(*W*) × 2.7 m(*H*), RNG *k-ε* turbulence model with enhanced wall treatment
Total supply airflow rate	0.1240 kg⋅s^−1^ (for *ach* = 3), 0.2480 kg⋅s^−1^ (for *ach* = 6), 0.3720 kg⋅s^−1^ (for *ach* = 9), 0.5374 kg⋅s^−1^ (for *ach* = 13), 285K (air temperature)
Each inlet airflow rate (0.6 m × 0.6 m)	0.031 kg⋅s^−1^ (for *ach* = 3), 0.0620 kg⋅s^−1^ (for *ach* = 6), 0.093 kg⋅s^−1^ (for *ach* = 9), 0.1343 kg⋅s^−1^ (for *ach* = 13), 285K (air temperature)
Each diffuser (0.6 m × 0.6 m)	Four supply diffusers, 4-way spread pattern, air supplied at an angle of 15° from the ceiling, adiabatic
Corridor (6 m × 2.7 m)	Outflow with flow rate weighting, 295K (backflow temperature), adiabatic, escape boundary type
Exhaust grille (0.5 m × 0.2 m)	Outflow with flow rate weighting, 295K (backflow temperature), adiabatic, escape boundary type, Exhaust air 0%, 10%, 50% of total supply air
Walls, ceiling, floor and beds	No-slip wall boundary, adiabatic, trap boundary type
Patients	Six patients, No-slip wall boundary, 23.3Wm^-2^ for each patient, trap boundary
Mouth of patient (0.05 m × 0.05 m)	Single shot release with an exhalation upward velocity of *v*_b_ = 50 ms^−1^ [47], *n*_*s*_ = 10,000 virus particles, density of bioaerosol particles *ρ*_*b*_ = 1,100 kgm^−3^
Species and their aerodynamic diameters	•MERS-*CoV* (0.167 ± 0.012 μm)

### 3.2 Airborne exposure to pathogens

The symptoms created by pathogens responsible for COVID-19, MERS-CoV, and other similar coronavirus infections bear resemblance to those of the common cold or influenza [48]. Thus, in certain instances, a patient with an infectious disease could be nursed in an open ward cubicle before receiving a diagnosis. During this time, the patient's exhalation activity, including sneezing, may release a significant number of pathogens into the ward air [49]. This could potentially lead to an outbreak of the disease [50,51]. This study reproduces a similar scenario by assuming the admission of patient 5 (source) to the ward at a random location as illustrated in Fig. 5. Therefore, an investigation is conducted into the exposure to pathogens for healthcare workers, visitors, and patients resulting from these occurrences.

The expelled pathogens will remain suspended in the air for a certain period, after which it most likely has three possible fates: deposition on surfaces, removal through the HVAC system, and inhalation by an individual. This study considers the risk of exposure to pathogens due to inhalation. Equation (4) accounts for an event where an individual gets exposed to infectious pathogens at their breathing height and gets infected by inhalation.

(4) $$N_{p}\left(t\right)=\int_{0}^{t}n\left(t\right)dt$$

**Fig. 4:**
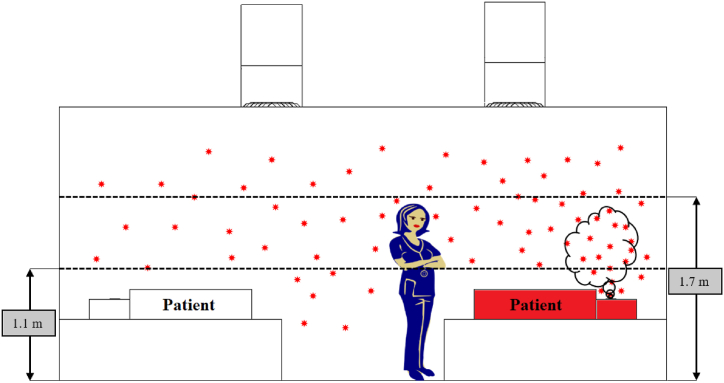
Breathing zone height and dispersion of infectious pathogens from an infected patient in an inpatient ward cubicle.

**Fig. 5:**
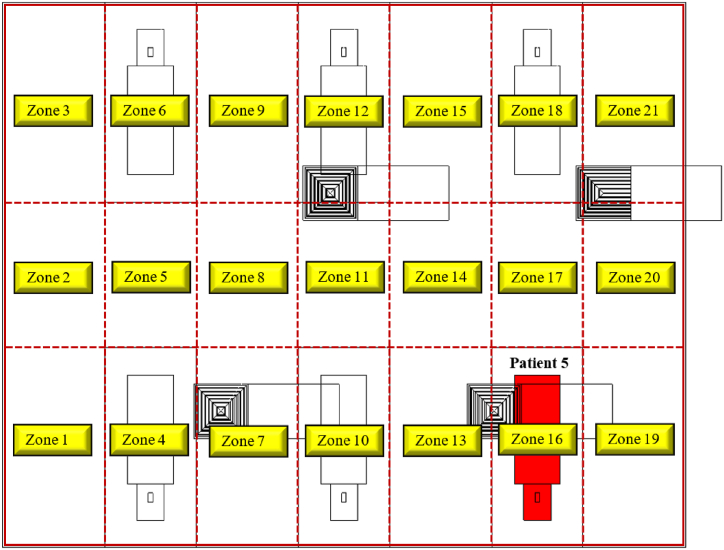
Breathing zones within the computational domain.

*N*_*p*_*(t)* is the total number of particles at time *t*, and *n(t)* is the rate of change of particles with respect to time. The integral is taken over the interval between when the particle is expelled and when a particle resides in the breathing zone. In this study, the breathing zone is taken as a height that varies between 1.1 m and 1.7 m as depicted in Fig. 4. The ward cubicle is segregated into several zones, as illustrated in Fig. 5 to estimate the spatial and temporal spread of the MERS-CoV droplet nuclei in the ward cubicle. To obtain the history of particles traversed through the several zones within the computational domain with respect to time, a region with prescribed coordinates is created in Ansys Fluent [52], and the discrete phase modelling (DPM) summary for the region is sorted for every second. After that, with the aid of a python programming code, the number of particles and residence time of each particle in each zone is determined from the DPM summary for every simulation scenario considered in this study.

### 3.3 Model validation

**Fig. 6:**
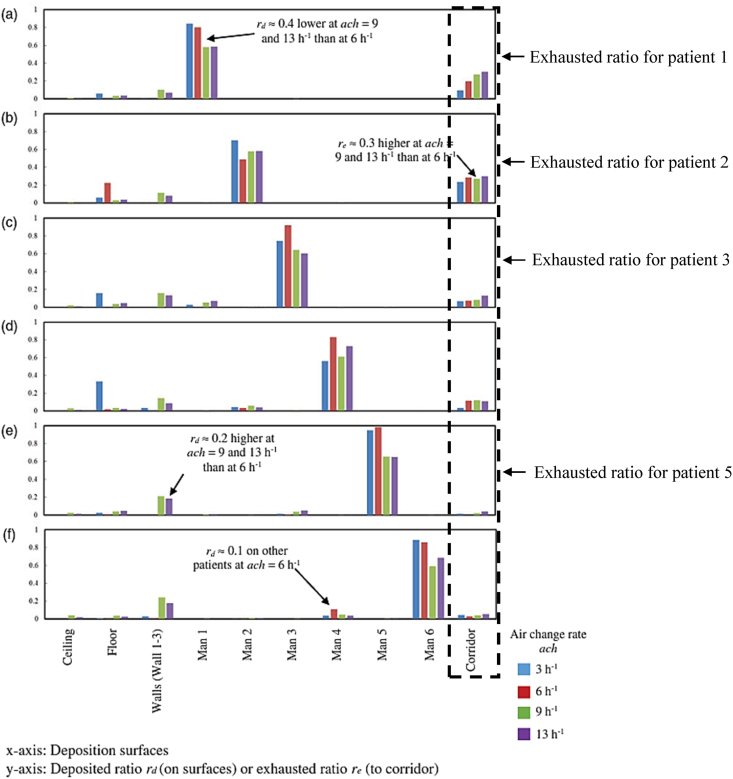
Deposited and exhausted ratios of MERS-CoV in an inpatient ward cubicle (Yu et al. [44]).

The model validation was conducted using a study done by Yu et al. [44]. Yu et al. [44] estimated the influence of air change rates on the dispersion and deposition mechanism of bioaerosols, specifically the MERS-CoV, which is emitted by a source patient in a mechanically ventilated inpatient ward cubicle. The study had analyzed the influence of source (patient) location on infection transmission under different air change rates by considering different emission locations within the ward cubicle. The validation of the computational fluid dynamics (CFD) simulation of this study is carried out by utilizing the particle exhausted ratio, which is extracted from Fig. 6(a–f). Equation (5) demonstrates that the exhausted ratio is determined by dividing the number of particles that are exhausted to the corridor by the overall number of particles that are expelled by the infected patient.

(5) $$r_{e}=\frac{n_{e}}{n_{s}}$$

**Fig. 7:**
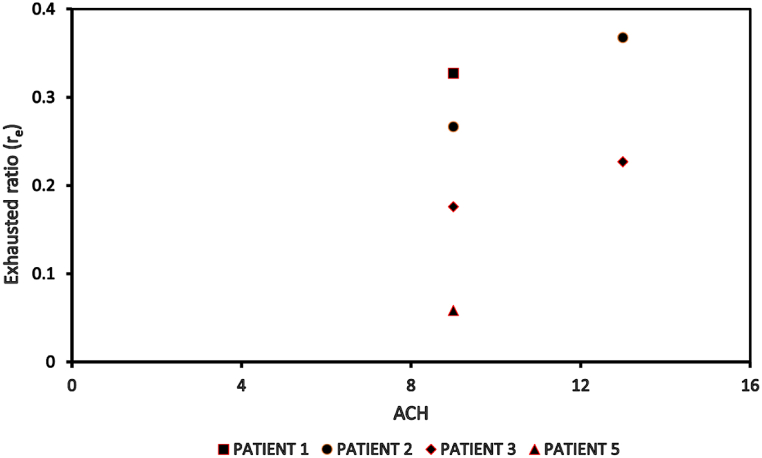
Exhausted ratio for different source (patient) at an air change rate of 9 h^−1^ and 13 h^−1^..

The exhausted ratio accounts for the possibilities of infection transmission from a ward cubicle to the corridor due to the spread of expelled particles from an infected patient to the corridor. According to Ref. [53], these particles could also move to neighboring spaces connected to the corridor. The CFD simulation for validation was conducted for the base case scenario of the ward cubicle without any local exhaust grille, as shown in Fig. 2 (a). As shown in Fig. 6, the exhausted ratio of four source patients, namely, patient 1, patient 2, patient 3 and patient 5 under an air change rate of 9 h^−1^ and 13 h^−1^ were taken for validation. As depicted in Fig. 7, the exhausted ratio documented for varying air change rates and source patients in this investigation aligns closely with the simulation findings of Yu et al. [44], with error less than 5%. This reflects the accuracy and reliability of the CFD simulation of this study to conduct further exploration.

## 4 Results and discussion

### 4.1 Airflow distribution and airflow pattern

**Fig. 8:**
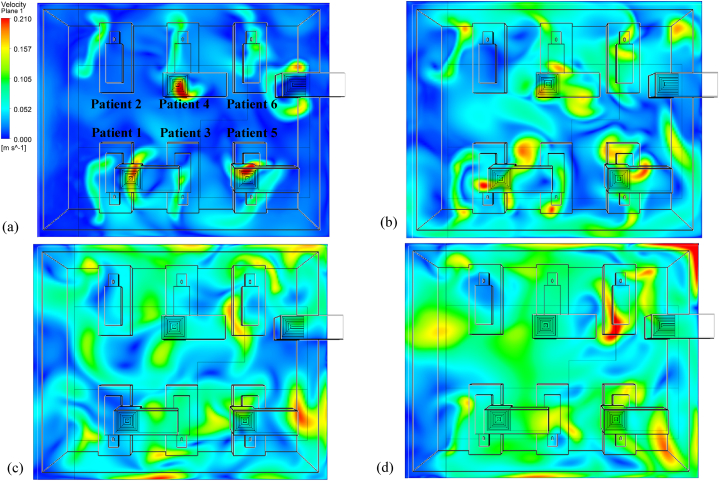
Air velocity distribution across a horizontal plane located at y = 1.35 m under (a) 3 ACH; (b) 6 ACH; (c) 9 ACH; and (d) 13 ACH.

The overall airflow distribution throughout the ward was generated using CFD simulations for four air change rates: 3 h^−1^, 6 h^−1^, 9 h^−1^, and 13 h^−1^. Fig. 8(a–d) depicts the airflow velocity contour at the height of 1.35 m. While stagnant air with a velocity as low as 0.01 ms^−1^ was seen on the opposite end of the corridor, moderate air velocity was detected around the beds at a low ACH of 3. However, there are regions of high air velocity directly beneath the supply diffuser. A gradual shift in the distribution of low-velocity zones is noticeable within the ward cubicle with an increased air change rate. There is a change in airflow velocity distribution in the aisles between the beds and the side walls as the ACH increases. However, the air velocity in the ward was relatively more evenly distributed at approximately 0.1 ms^−1^ at the highest ACH of 13. Yet stagnant air zones were observed near patients 1 and 2, closer to the corridor. The regions close to the source patient (patient 5) and patient 6 were observed to have velocities as high as 0.2 ms^−1^. With an increase in ACH, there was anticipated to be having more air mixing, which could aid in the long-distance dispersion of airborne MERS-CoV particles. However, the location of the air supply diffuser and the velocity of the supply air could play a significant role in the transmission of infectious pathogens.

**Fig. 9:**
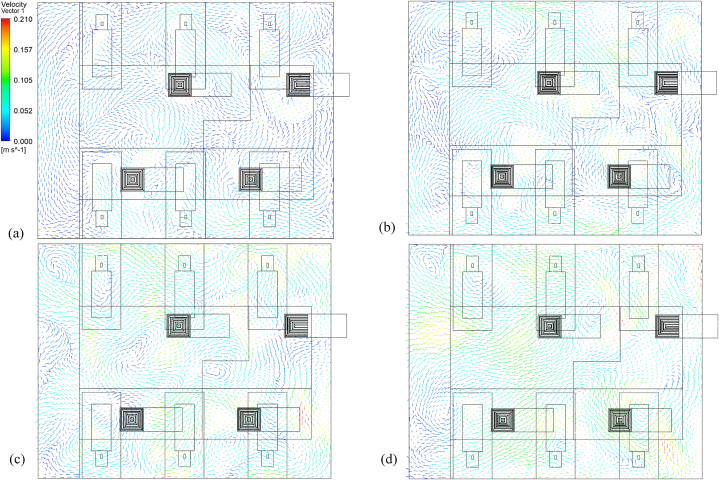
Airflow pattern across a horizontal plane located at y = 1.35 m under (a) 3 ACH; (b) 6 ACH; (c) 9 ACH; and (d) 13 ACH.

Due to positive pressure in the ward, the velocity vectors in Fig. 9(a–d) indicate a general airflow direction from the ward interior towards the corridor. The formation of eddies adjacent to the beds due to the turbulent flow and physical obstructions would affect the dispersion and deposition of particles within the cubicle. The airflow around the source patient at an air change rate of 3 h^−1^ shows that it could assist in the movement of particles to zones located in proximity. The bedside zone 13 could receive most of the expelled particles within seconds of their release from the source patient. At the same time, the movement of air from the source patient could assist in putting the adjacent patient (patient 3) at a higher risk for infection transmission than others while the patient is sitting upright in their bed. The movement of air towards the aisle at the centre of the cubicle that separates two sections of patients would also cause infection transmission to users. The formation of recirculation zones in the ward centre between the source and patient 6 would trap infectious pathogens for an extended period before their eventual removal. At an ACH of 6, the air velocity distribution generates more mixing within the ward cubicle. Yet, the formation of recirculation zones above patients 3 and 4 could build up infectious pathogens in these regions. With an air change rate of 9 h^−1^, mixing is augmented. The airflow distribution is observed to become more uniform across different zones within the cubicle at 13 h^−1^. Mixing air streams across both halves of the ward cubicle is observed. The basic airflow pattern in the ward is from the interior to the corridor, with the establishment of recirculation zones being minimized.

### 4.2 Spatial and temporal distribution of particles

The distribution of particles in different zones with regards to time is discussed. To improve the clarity of results and their associated insights, the results are presented by bringing the segregated zones in the ward under three main zones as shown in Table 2.

**Table 2:** Allocation of zones.

Patient Zones	Bedside Zones	Aisle zones
4,6,10,12,16,18	1,3,7,9,13,15,19,21	2,5,8,11,14,17,20
		

#### 4.2.1 Patient zones

**Fig. 10:**
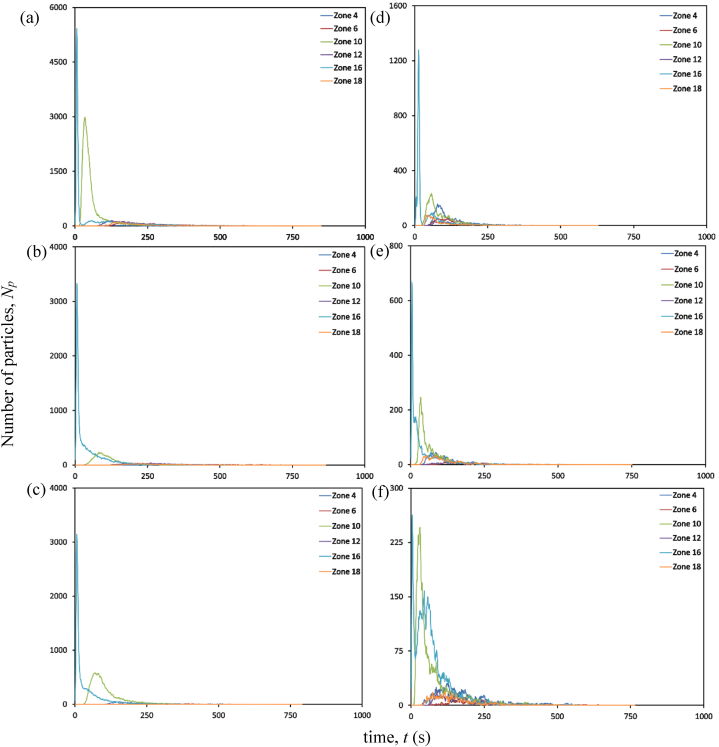
Particle distribution at patient zones at different air change and exhaust flow rates (a) ACH 3; (b) ACH 3 and exhaust flow rate 10%; (c) ACH 3 and exhaust flow rate 50%; (d) ACH 13; (e) ACH 13 and exhaust flow rate 10%; (f) ACH 13 and exhaust flow rate 50%.

At an air change rate of 3 h^−1^, the maximum concentration of particles remaining suspended in air is observed primarily in the zone (zone 16) directly above the source patient, as indicated in Fig. 10 (a). The particles reach this zone as soon as the source patient sneezes. Within 10 s, over half of all particles released during sneezing reach this region. After 10 s, there is a progressive decrease in the accumulation of particles in Zone 16. After 30 s, there is an increase in particle accumulation in the zone directly above the adjacent patient. This trend could arise from the movement of particles under the influence of airflow from Zone 16 to other locations within the ward. Over time, the patient zone adjacent to the source patient will likely be the most hazardous. The corridor-directed airflow distribution pattern plays a crucial role in the passage of particles from the contaminated source patient zone to the adjacent patient zone. The decrease in particle accumulation in zones 10 and 16 marks the beginning of the presence of particles across other patient zones. With the increased air change rate of 13 h^−1^, the number of particles reaching the breathing zone directly above the source patient is drastically reduced, as indicated in Fig. 10 (d). This could be attributed to the momentum of airflow guided by ceiling-mounted diffusers. This reduction in the number of particles would reduce the number of particles carried to different patient zones in the ward. This is significant compared to the results achieved with a lesser flow rate at 3 h^−1^.

A significant reduction in particle concentration is evident with installing local exhaust grilles near the source patient, notably in Zone 16, as shown in Fig. 10 (b) and (c). Installing the exhaust grille is beneficial, as seen by the decrease in suspended particles in the patient zones. After peaking at Zone 16, the number of particles declines within 30 s. Immediately after its decline, particle increase is seen in its adjacent zones. However, the most significant reduction in the number of particles remaining airborne is achieved with the increase in exhaust flow rate from 10% to 50% at a higher ACH (13 h^−1^), as indicated in Fig. 10 (e) and (f). Consequently, this particle decrease results in less particle migration to other zones. A local reduction in the number of particles reduces the availability of infectious pathogens to cause an infection.

#### 4.2.2 Bedside zones

**Fig. 11:**
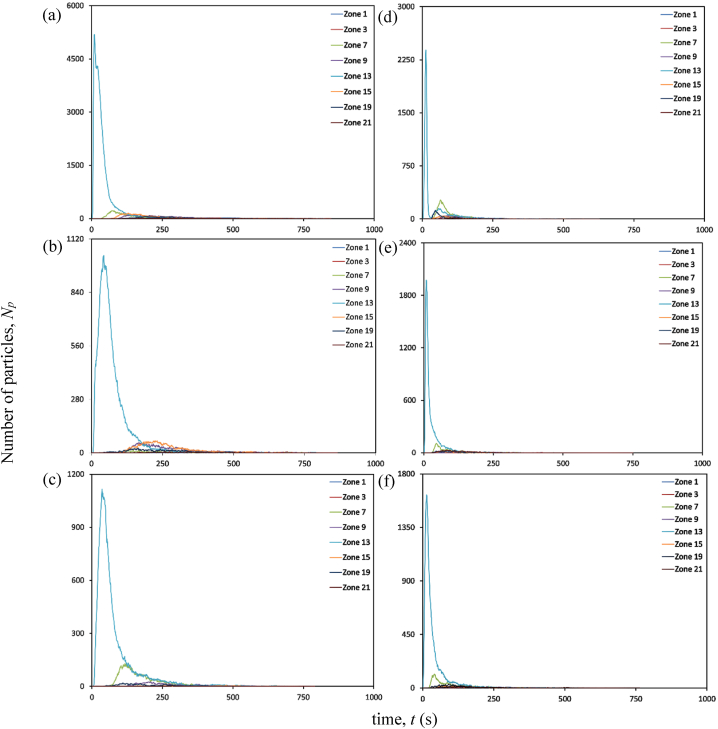
Particle distribution at bedside zones at different air change and exhaust flow rates (a) ACH 3; (b) ACH 3 and exhaust flow rate 10%; (c) ACH 3 and exhaust flow rate 50%; (d) ACH 13; (e) ACH 13 and exhaust flow rate 10%; (f) ACH 13 and exhaust flow rate 50%.

The exhaled particles in the ward are transported and dispersed due to the airflow distribution established in the room. As indicated in Fig. 11 (a), at an air change rate of 3 h^−1^, particles in the bedside zone are first noticed in Zone 13, near the source patient. The particles reach this zone as soon as the source patient sneezes. Within 11 s, this zone occupies the highest number of particles; after that point, the number of particles staying suspended in Zone 13 decreases gradually. The accumulation of many particles in Zone 13 shortly after its emission can be linked to the airflow direction and the source patient's proximity. As particles in this zone decrease, particles migrate to other zones. Within 60 s after particle emission, particles are detected in zones 7, 15, and 21 that are located farther from the source patient. There is a gradual shift in the distribution of particles within the ward over time. Nonetheless, the particles reaching these zones are significantly lower than in the zone around the source patient. This is an important insight to consider in the spread of infection. The transfer of infectious pathogens via the air from their source to other sites may result in the transmission of infectious diseases to other ward users, including healthcare professionals, visitors, and patients.

The effectiveness of an exhaust grille positioned near the source patient is analyzed. An increase in the number of particles is observed in the area immediately adjacent to the source patient, a situation analogous to the absence of a local exhaust grille. In Zone 13, the maximum accumulation of particles is observed 45 s following the emission of particles from the source patient. However, with a local exhaust grille and an exhaust flow rate of 10%, the number of particles suspended in air is dramatically reduced compared to the scenario without an exhaust grille, as indicated in Fig. 11 (b). A reduction in Zone 13 signifies the beginning of the movement of particles to other zones within the ward. The accumulation of particles in Zones 9 and 15 is higher than in other zones after 100 s of the particle release. Although, after 500 s, the number of particles in most zones reduces to less than 10.

After the number of particles in the air peaks at 40 s, there is a progressive decrease in the accumulation of suspended particles. A gradual increase in particle accumulation is seen in Zone 7. Yet, when the particle accumulation in Zone 7 peaks at 117 s, the particles decrease gradually. Under an exhaust flow rate of 50%, the profile of particle build-up in Zone 13 is nearly identical to the case with an exhaust flow rate of 10%. However, at an exhaust flow rate of 50%, the transport of particles to other areas of the ward is severely constrained, as seen in Fig. 11 (c).

One of the main advantages of adopting an air change rate of 13 h^−1^ is that there are fewer particles in the breathing zone than with an air change rate of 3 h^−1^, as observed In Fig. 11 (d). The number of particles accumulating in the breathing zone within seconds of discharge is cut in half compared to the scenario with an air change rate of 3 h^−1^. After 300 s, the quantity of particles remaining in the bedside breathing zones is dramatically reduced. A modest particle increase is observed in Zones 7 and 19 shortly after a drop in suspended particles in Zone 13.

Including an exhaust grille reduces the presence of infectious particles remaining suspended in the bedside breathing zone. A decline in the accumulation of particles is noted for different bedside breathing zones within the ward, with an exhaust flow rate of 10% and 50%. After 200 s, fewer than 10 particles are observed to be suspended in the air, as indicated in Fig. 11 (e) and (f). The maximum particle accumulation in the breathing zone is observed in Zone 13 after particle emission. This behaviour is identical in all the circumstances presented in this study, with the primary variable being the number of accessible particles that can promote airborne transmission. The most significant presence of particles in the breathing zones is observed without any local exhaust grille. Hence, the upgradation of the ward with the installation of a local exhaust grille would be an effective solution. Within 200 s, an exhaust grille with an air change rate of 13 h^−1^ tends to make the breathing zones within the bedside less contaminated. This is exceptional compared to the other ventilation techniques considered in this study.

#### 4.2.3 Aisle zones

**Fig. 12:**
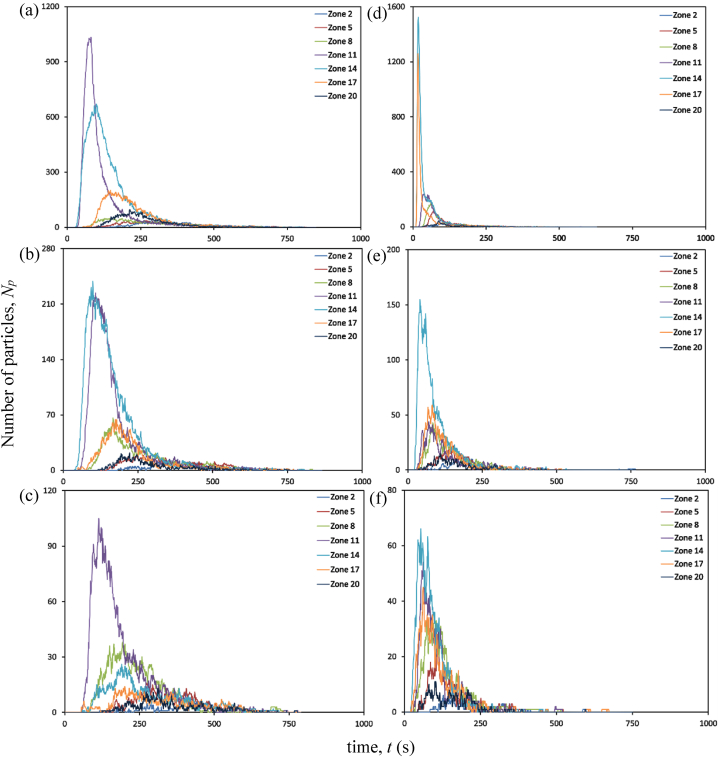
Particle distribution at aisle zones at different air change and exhaust flow rates (a) ACH 3; (b) ACH 3 and exhaust flow rate 10%; (c) ACH 3 and exhaust flow rate 50%; (d) ACH 13; (e) ACH 13 and exhaust flow rate 10%; (f) ACH 13 and exhaust flow rate 50%.

At an air change rate of 3 h^−1^, as indicated in Fig. 12 (a), an increase in particle accumulation is experienced in Zone 11, followed by Zone 14 after 30 s of particle release. The highest accumulation is observed in Zone 11, which peaks at 80 s before gradually decreasing. The reduction in particle accumulation in these two zones marks the rise in particles across other zones. The proximity of Zones 11 and 14 to the source patient may be one of the primary causes of the increase in particle counts.

Analyses are conducted to determine the efficacy of a 10% exhaust flow rate on particle distribution. The concentration of particles is higher in Zones 11 and 14, as seen in the case with no local exhaust grille. However, a reduction in the number of particles remaining suspended in individual breathing zones is noticed with time, as shown in Fig. 12 (b). With an increase in exhaust flow rate at an air change rate of 3 h^−1^, the number of particles staying in the breathing zone decreases even further, as indicated in Fig. 12 (c). With time, the presence of particles is discernible in all zones of the aisle. An increase in exhaust flow rate to 50% minimizes the particle build-up in the breathing zones of the aisle, and a considerable decrease is observed in Zone 14.

Under an air change rate of 13 h^−1^, Zones 14 and 17 have the highest particle build-up within the aisle, as shown in Fig. 12 (d). The maximum accumulation tends to occur within 20 s of particle emission from the source patient. However, after peaking, it reduces substantially within 50 s. The reduction in these zones marks the growth in other zones across the aisle, with zones 11 and 8 experiencing a considerable increase. Nonetheless, the build-up diminishes dramatically within 100 s in these zones. The aisle zones are clear of any infectious particles within 300 s after its discharge, as indicated in Fig. 12 (d).

With the installation of a local exhaust grille, the accumulation of suspended particles in the aisle is significantly reduced. A notable decrease in particle accumulation is evident in zones 11, 14 and 17. Earlier, these zones accumulated particles in settings with no local exhaust grille. The presence of suspended particles reduces significantly with exhaust flow rates of 10% and 50%, as indicated in Fig. 12 (e) and (f). Zone 14 appears to be the site with the most significant exposure to infectious particles in all scenarios represented under the air change rate of 3 and 13 per hour. The other zones are also exposed to particles expelled from Zone 16, although the number of particles is relatively low. Installing a local exhaust grille provides the benefit of dramatically reducing the availability of particles causing airborne disease transmission. In addition, it is essential to note that combining a high exhaust flow rate with a high ACH is preferable to a low ACH, as the former combination would decontaminate the space more quickly than the latter.

**Fig. 13:**
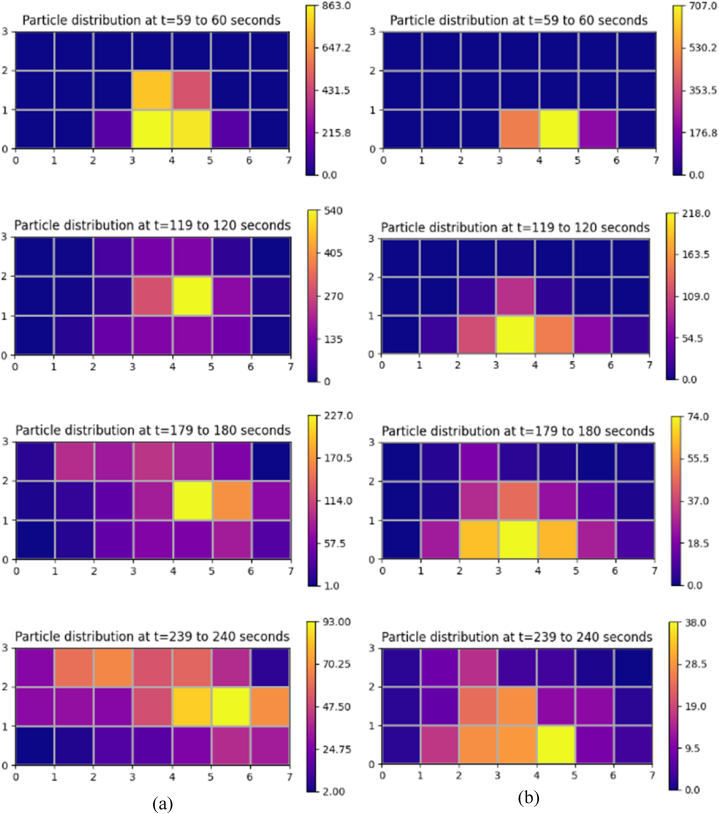
Spatial and temporal distribution of particles in different zones within the breathing height under different conditions (a) ACH 3 (b) ACH 3 and exhaust flow rate 50%.

**Fig. 14:**
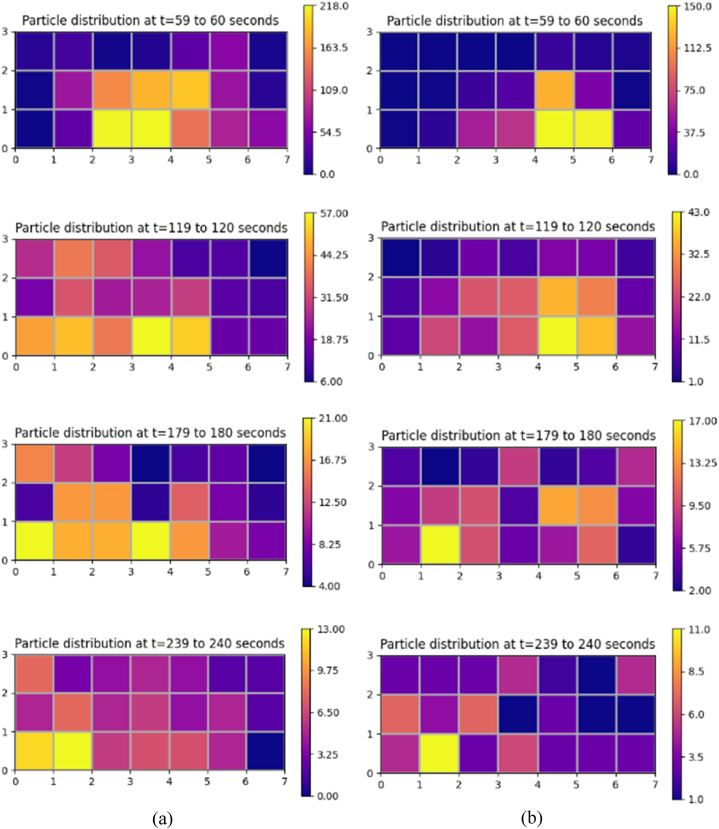
Spatial and temporal distribution of particles in different zones within the breathing height under different conditions (a) ACH 13 (b) ACH 13 and exhaust flow rate 50%.

Fig. 13 (a, b) and Fig. 14 (a, b) depicts a particle distribution plot to aid in the comprehension of the dispersion of particles throughout the ward. It represents particles' spatial and temporal distribution in various zones under the different air changes and exhaust flow rates addressed in this study. One minute after its release, it could be seen from the plot that the spread of particles is initially restricted to zones near the source patient. However, as time passes, the particles start to move away from its source towards other locations within the ward. The plot provides us an insight that under the effect of airflow, the particles could migrate several meters away from their point of origin, resulting in the transfer of infectious diseases within the ward. However, due to the effectiveness of the local exhaust grille, the number of particles available to flow across zones and induce infection transmission is significantly reduced. An increased ventilation rate complimented with an exhaust flow rate of 50% through a local exhaust grille is shown to provide a better performance in providing a localized control to restrict infection transmission. Our work has devised an effective ventilation strategy that provides enhanced protection to ward users against airborne dispersion of infectious pathogens without the need for massive revamp of the entire inpatient ward facility.

## 5 Limitations and future work

The current study provided important insights into the role of ventilation strategy in infection transmission mechanisms in a hospital inpatient ward cubicle. Nonetheless, it is essential to note that this study had a few limitations. The impact of the movement of staff, patients, and visitors should have been addressed. Nevertheless, this factor can cause disturbances in airflow and particle distribution. Few studies, however, indicate that its effect is temporary and considerably less significant than ventilation [54,55]. Moreover, in this study, 100% outside air was assumed in every simulation. Therefore, it is further necessary to consider the effectiveness of the suggested ventilation strategies while also taking air recirculation into account. In addition, the impact of variation in the number, location, and size of supply diffusers and exhaust grilles should have been considered. These variations in design would provide additional insights into the transmission mechanism of infectious diseases. These scenarios, as discussed earlier, may or may not enhance the current knowledge gained from this study. Nonetheless, it is essential to evaluate the efficacy of these scenarios so that every situation is explored for the betterment of healthcare facilities. The current study's findings will aid in defining the design space that requires further investigation.

## 6 Conclusion

The possibility of airborne transmission of infection caused by the emission of pathogens by exhalation activity, such as sneezing of an undiagnosed patient with infectious diseases admitted to general inpatient wards, is a major concern. General inpatient wards occupy a large portion of a hospital's floor space. However, the ventilation strategy for this facility needs to be more well-established compared to operation theatres, intensive care units and isolation rooms. The role of ventilation in the infection transmission mechanism in indoor environments is indisputable. Hence, this research investigated the influence of ventilation strategies in terms of air change and exhaust flow rates on the exposure of individuals to infectious pathogens in breathing zones within an inpatient ward cubicle. Exhalation activity like sneezing from a source patient generates numerous infectious pathogens. The breathing zone above the source patient will receive most of the particles shortly after their emission. This behaviour was identical in all the scenarios considered in this study. The decline in the number of suspended particles in the air in the zone above the source patient marks the migration of particles to other zones within the ward cubicle. Within a few seconds of particle emission, the bedside zone and zone above the adjacent patient close to the source patient are deemed more hazardous. With time, the transport of particles to other zones occurs. The migration of particles to aisle and patients located opposite to the source patients presents possibilities for cross-infection. The transport of particles to locations several meters away from their source reveals its ability to cause infection outbreaks within the facility. The infected ward users in the early transmission stages could cause massive community outbreaks through their day-to-day interactions. However, a key difference while adopting a lower ACH (3 h^−1^) and higher ACH (13 h^−1^) as used in this study was that the latter had a significantly lower number of suspended particles in the breathing zone than the former. The installation of exhaust grilles was advantageous, as it was evident that combining a higher air change rate (ACH) with a high exhaust flow rate (50 percent of supply air) through a local exhaust grille would result in a dramatic reduction of suspended particles within the breathing zone, further mitigating the risk of pathogen exposure for ward users. Thus, while concluding this section by reiterating the importance of establishing effective ventilation strategies to reduce the risk of infection transmission in presumably low-risk areas such as general inpatient wards, this study proposes the use of higher ACH in conjunction with a high exhaust flow rate through a local exhaust grille positioned close to patients.

## Data availability statement

Data included in article/supp. Material/referenced in article.

## Funding

This work was supported by a grant from the Collaborative Research Fund (CRF) COVID-19 and Novel Infectious Disease (NID) Research Exercise, Research Grants Council of the Hong Kong Special Administrative Region, China (Project no. PolyU P0033675/C5108-20G).

## CRediT authorship contribution statement

**Manoj Kumar Satheesan:** Writing – original draft. **Tsz Wun Tsang:** Methodology, Data curation. **Ling Tim Wong:** Supervision. **Kwok Wai Mui:** Supervision, Project administration, Conceptualization.

## Declaration of competing interest

The authors declare the following financial interests/personal relationships which may be considered as potential competing interests: Kwok-Wai Mui reports financial support was provided by Research Grants Council of the Hong Kong Special Administrative Region, China.
